# Neonatal Outcomes in Maternal Depression in Relation to Intrauterine Drug Exposure

**DOI:** 10.3389/fped.2019.00309

**Published:** 2019-07-26

**Authors:** Silvia Corti, Paola Pileri, Martina I. Mazzocco, Chiara Mandò, Anna F. Moscatiello, Dario Cattaneo, Stefania Cheli, Sara Baldelli, Laura Pogliani, Emilio Clementi, Irene Cetin

**Affiliations:** ^1^Department of Mother and Child, ASST Fatebenefratelli-Sacco, University of Milan, Milan, Italy; ^2^Clinical Pharmacology Unit, Department of Biomedical and Clinical Sciences, Consiglio Nazionale delle Ricerche Institute of Neuroscience, University of Milan, Milan, Italy; ^3^IRCCS E. Medea Institute, Bosisio Parini, Italy

**Keywords:** SSRI, pharmacogenetics, poor neonatal adaptation syndrome, newborns, pregnancy, depression

## Abstract

**Background:** SSRIs (Selective Serotonin Reuptake Inhibitors) are the most useful drugs to treat depression during pregnancy. Intrauterine exposure to SSRIs may increase the risk of growth restriction, preterm birth and neonatal complications. However, advantages in treating depression seem to exceed potential drug side effects in respect un-treated depression. SSRIs undergo extensive hepatic first-pass metabolism with the involvement of several cytochrome P450 (CYPs) enzymes. Genetic polymorphisms may influence the expression and activity of CYPs genes. The first aim of this study was to evaluate neonatal outcomes in depressed mothers exposed to SSRIs during pregnancy. SSRIs pharmacogenetics was also evaluated in a subset of mothers and fetuses.

**Methods:** In this case-control study, *cases* (*n* = 42) were Caucasian women with a diagnosis of depression and/or anxiety, treated with SSRIs for the whole pregnancy. *Controls* (*n* = 85) were Caucasian women without a psychiatric diagnosis and not exposed to SSRIs during pregnancy. Exclusion criteria for both groups were other psychotropic drugs, anti-epileptics, drug of abuse, alcohol addiction, maternal or fetal infectious diseases, fetal/neonatal chromosomal genetic abnormalities. Maternal and fetal blood samples were obtained at delivery to analyze genotype in 33 *cases*.

**Results:** The population was homogenous for demographic, anthropometric, socio-economic and obstetric variables except for smoking and mean hemoglobin values before delivery. Obstetric features were comparable. Newborns exposed to SSRIs during fetal life were significantly more likely to be Low Birth Weight (LBW) (birth weight <2,500 g) (*p* = 0.01), had significantly lower mean Apgar scores at 1' (*p* = 0.006) and at 5' (*p* = 0.023) and worse Apgar distribution at 1' (*p* = 0.017) and at 5' (*p* = 0.013). Fifty-six percent of newborns presented one or more symptoms consistent with poor neonatal adaptation syndrome (PNAS). Pharmacogenetic analysis at delivery did not show significant differences in the frequencies of obstetric or neonatal complications in relation to polymorphisms.

**Conclusions:** We found that newborns exposed to SSRIs are at increased risk of poor neonatal outcomes in terms of low birth weight, low Apgar scores and, clinically, poor neonatal adaptation syndrome. Preliminary pharmacogenetic analysis showed that the degree of CYPs alterations, that depends on polymorphisms, may influence neonatal outcomes.

## Introduction

Recent studies have shown that 2–9% of women in the Western world are prescribed antidepressants (AD) during pregnancy (1). Intrauterine exposure to antidepressant drugs may have adverse consequences for birth outcome and child development (2, 3). Antidepressants use during pregnancy can lead to increased risk of miscarriage, congenital cardiac malformations, preterm birth (PTB), persistent pulmonary hypertension of the newborn (PPHN), and poor neonatal adjustment syndrome (PNAS) (4, 5). Multiple studies have reported an increased risk of PTB (OR ranging from 1.5 to 2) with prenatal exposure to antidepressants (6). It has been reported a possible increased risk of delayed neurobehavioral development in children (7). Several recent systematic reviews and meta-analyses emphasize that there are minimal definitive conclusions to guide treatment (8). In this view it is important to evaluate potential neonatal risks such that pregnant women can be provided with better counseling and midwives and pediatricians alerted at delivery.

Exposure to untreated depression and stress during pregnancy may have negative consequences for birth outcome and child development (9). Suicidal ideation also can be elevated in pregnant women with depression, representing a real risk for the life of both the mother and the fetus (10, 11).

Selective serotonin reuptake inhibitors (SSRIs) are the drug of choice, for their safety profile, in terms of fewer metabolites, higher protein binding to decrease placental passage, and fewer interactions with other medications (12). Sertraline, paroxetine, fluoxetine, fluvoxamine citalopram, and escitalopram are included in this group.

Results of studies examining the effects of antidepressants exposure on birth outcomes and child development have been inconsistent, since the contribution from the underlying mood disorder and the associated behaviors (e.g., poor prenatal health behaviors, concomitant medications, smoking, substance abuse, and obesity) may be confounding variables (6).

In pregnancy there are changes in drugs absorption, distribution, metabolism, and elimination. Therefore, antidepressants dosage should be increased to maintain a therapeutic effect (13, 14). Fetal exposure to antidepressants occurs through umbilical cord blood flow, placental transport, and absorption from amniotic fluid. This is influenced by the cord-to-maternal ratio of the antidepressant level, their half-life and peak levels, genetic polymorphisms of drug metabolism enzymes and transporter proteins, and the unbound fraction of antidepressant in the fetus (15).

SSRIs undergo extensive hepatic first-pass metabolism with the involvement of several cytochrome P450 (CYPs) enzymes. Expression of CYPs genes is influenced by a combination of factors including genetic polymorphisms, drug-drug interactions, patients' age. Distinct CYPs pharmacogenetic phenotypes, derived from polyallelic mechanisms, have been associated to the dose requirement, drug efficacy and occurrence of severe toxicities (16, 17).

We recently reported preliminary observations on the contribution of individual pharmacogenetics of SSRIs on infants' outcome. We also estimated the umbilical/maternal plasma SSRI concentration ratio at delivery (18).

The primary aim of this study was to evaluate neonatal outcomes in depressed mothers exposed to SSRIs during pregnancy. We also evaluated SSRIs pharmacogenetics in a subset of mothers and fetuses.

## Methods

This was a prospective, observational, and experimental case-control study. It was performed from January 2011 to May 2016 in the Unit of Obstetrics-Gynecology, Sacco Hospital, Milan in collaboration with the Units of Neonatology and Pharmacology. The Medical Ethics Committee of Sacco Hospital approved the study protocol and the consent procedure. All women gave their written informed consent for their own and on behalf of their newborns.

Forty-nine *cases* and 100 controls were prospectively enrolled. *Cases* were Caucasian women with a diagnosis of depression and/or anxiety in treatment with selective serotonin reuptake inhibitors (SSRIs) before conception. Venlafaxine was considered as an SSRI if being dosed <200. We also included in the study women taking anxiolytics such as benzodiazepines, if needed.

The control group included Caucasian women, not exposed to psychotropic medications before and during pregnancy. Two unexposed subjects per one SSRI-exposed subject were randomly selected from the delivery register of the hospital, by taking the first two deliveries immediately after each SSRI case.

The exclusion criteria were the use of other psychotropic drugs (besides SSRIs or sporadic benzodiazepines), anti-epileptic drugs, drug of abuse or alcohol addiction, maternal or fetal infectious diseases, fetal/neonatal chromosomal genetic abnormalities. Women who stopped taking medication before or during labor were excluded from the study.

Out of the 48 enrolled *cases*, two were lost to follow-up, two were excluded because in treatment with antipsychotics or antidepressants other than SSRI, and two had not yet delivered at the end of the study, leaving 42 *cases* with 42 newborns.

All *cases* were followed in a prospective way by our multidisciplinary team, that checked drug compliance, psychologic clinical control and maternal and fetal well-being at each visit during the whole pregnancy up to delivery.

Maternal and fetal data of the control group were collected from the registers and databases of Sacco Hospital. Forty-two *cases* were compared to 85 controls for neonatal outcomes.

We evaluated and compared pregnancy complications such as Gestational Diabetes, Intrauterine Growth Restriction (IUGR), Pregnancy Induced Hypertension, Cholestasis of pregnancy, Preterm Premature Rupture of Membranes (pPROM), Hyperemesis Gravidarum, Polyhydramnios, Oligohydramnios, Anhydramnios, and delivery outcomes in the two groups, including variables such as mode of delivery, urgent cesarean section, vacuum-assisted vaginal delivery, induction of labor, epidural anesthesia, meconium-stained amniotic fluid third degree.

We evaluated neonatal anthropometric characteristics such as birthweight, neonatal sex, gestational age at birth, Apgar score at 1 and 5 min, umbilical artery pH and base excess (BE) at blood gas analysis on cord blood. Neonatal complications considered in this study were Intrauterine Growth Restriction (IUGR), preterm birth, low birth weight (LBW), Small for Gestational Age (SGA), Apgar scores at 1' and 5' lower than 7, neurologic symptoms, Respiratory Distress Syndrome (RDS), Hypoglycemia, malformations, access to Neonatal Intensive Care Unit, and Poor Neonatal Adjustment Syndrome (PNAS).

SSRI's pharmacogenetics were evaluated by analyzing *cases'* maternal and blood samples that were collected at delivery. Fetal samples were collected from cord blood. Immediately after withdrawal, each blood sample was collected in a vial with sodium citrate and kept on ice until centrifugation. Samples were stored at −20°C for further genotyping at the centralized pharmacogenetics laboratory of the Pharmacology Unit of Sacco Hospital.

**Figure 1:**
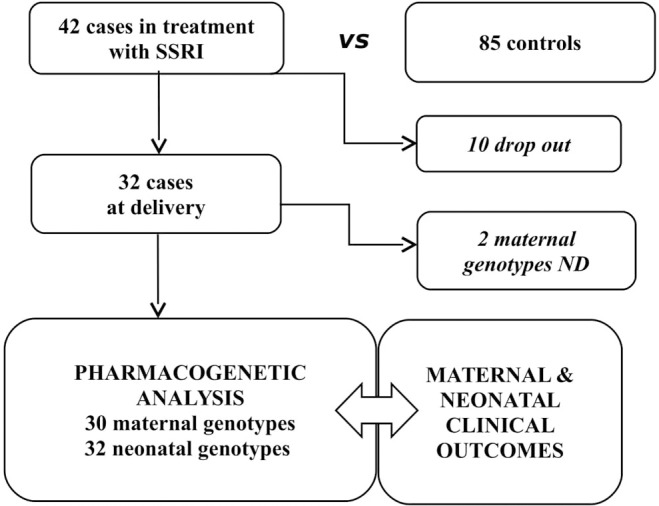
Study population.

Of 42 *cases*, 35/42 valid samples were collected for genotyping, and 32 maternal and neonatal samples were included in the pharmacogenetic analysis focused at the time of delivery. Two mothers had some missing data (nd) relatively to genotype (Figure 1).

Genomic DNA was isolated from peripheral blood cells using an automatic DNA extraction system (Maxwell^®^ 16 System, Promega, Madison, WI, USA) according to the manufacturer's instructions. Polymorphisms were determined by Real-Time PCR using LightSNiP^®^ (TIB-MolBiol, Berlin) or TaqMan^®^ assay (Thermo Fisher Scientific, Waltham, MA, USA) according the manufacturer's instructions. We selected and evaluated the functional variants mapping in CYP2D6 (^*^3,^*^4,^*^5,^*^6, rs1080985 promoter variant, gene duplication) for paroxetine, venlafaxine and citalopram, CYP2C19 (^*^2, ^*^3, ^*^17) for sertraline and citalopram, CYP2B6^*^6 for sertraline, CYP3A4^*^22 and CYP3A5^*^3 for citalopram (19).

### Statistical Analysis

Results are reported as mean values ± standard deviation (SD).

Maternal and fetal characteristics were compared between groups using independent-samples *t*-test. Levene's test for equality of variances was performed to assess whether data were obtained from populations of equal/unequal variances. Chi-square analyses were performed to compare characteristics expressed as frequencies. Analyses were considered significant when *p* < 0.05. Tests were performed by statistical package SPSS (IBM-SPSS-Statistics 21.00, Armonk, NY).

## Results

One hundred twenty-seven pregnant women were studied 42 *of whom* in treatment with SSRIs (cases) and 85 without (controls). The control population showed similar demographic, anthropometric and socio-economic features, except for smoking (Table 1); higher rates of smoking among depressed women compared to women without a psychiatric diagnosis were previously reported. We did not find relevant differences in major comorbidities prevalence, such as hypertension or diabetes mellitus.

**Table 1:** Characteristics of the population.

	**Controls (*n* = 85)**	**SSRIs (*n* = 42)**	***p-*value**
Age (years)	32.7 ± 5.9	34.4 ± 5.2	NS
Pregestational Body Mass Index (Kg/m^2^)	23.4 ± 4.5	23.9 ± 4.9	NS
Gestational weight gain (Kg)	11.8 ± 5.0	12.6 ± 4.7	NS
Smoking (any quantity)	3.0 (3.5%)	7.0 (16.7%)	0.012
Marital status			NS
Married	57 (67.1%)	29 (69.0%)	
Not married	28 (32.9%)	13 (31.0%)	
Single	0	0	
Occupational status			NS
Occupied	60 (70.6%)	33 (78.6%)	
Not occupied	25 (29.4%)	9 (21.4%)	
Parity			NS
Primiparous	30 (35.3%)	19 (45.2%)	
Multiparous	55 (64.7%)	23 (54.8%)	
Multiple pregnancy (twins)	1 (1.2%)	1 (2.4%)	NS
Onset of pregnancy			NS
Spontaneous	81 (95.3%)	41 (97.6%)	
ART	4 (4.7%)	1 (2.4%)	

*Data are presented as mean (± SD) or as number of women (%)*.

*p: NS = not significant, < 0.05 = significant*.

*SSRIs, selective serotonin reuptake inhibitors*.

*ART, assisted reproductive technology*.

In the SSRIs population depressive disorder, including both major and minor ones, accounted for 43% of the cases, anxiety disorders, including generalized anxiety, panic and obsessive-compulsive disorders for 26%, mixed anxiety-depressive disorder for 26%, and bipolar ones for 5%. Sertraline (40%) and paroxetine (31%) were the two most used molecules accounting together for more than 80%. Nineteen percent of patients had also received prescription for an anxiolytic drug belonging to the family of benzodiazepines for sporadic use.

**Figure 2:**
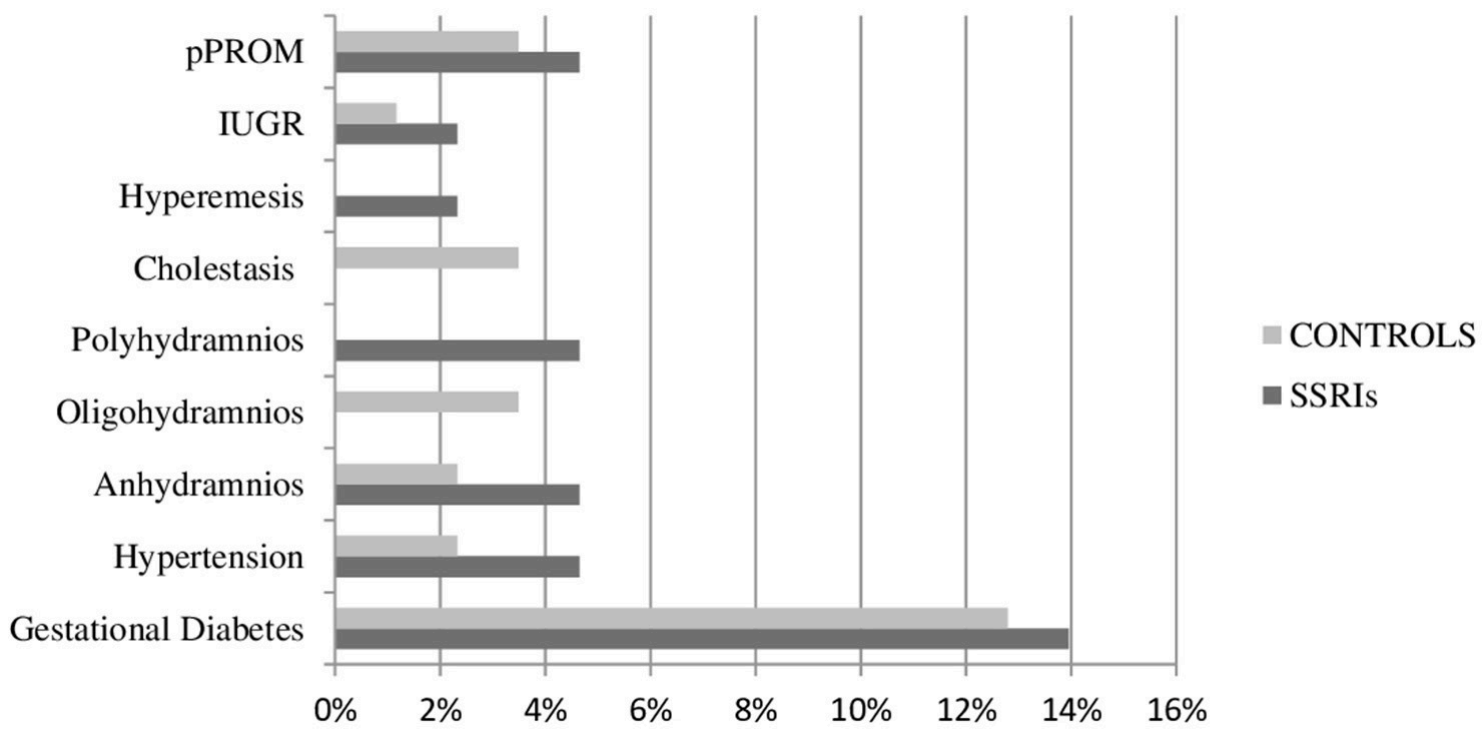
Pregnancy complications in the study population. Data are presented as percentage of women (%) on the population. SSRIs, selective serotonin reuptake inhibitors; IUGR, intrauterine growth restriction; pPROM, preterm premature rupture of membranes.

Pregnancy complications and obstetric outcomes were similar in the two groups, as shown in Table 2 and Figure 2.

**Table 2:** Pregnancy and obstetric outcomes.

	**Controls (*n* = 85)**	**SSRIs (*n* = 42)**	***p-*value**
Gestational age at delivery (weeks)	39.4 ± 1.3	39.0 ± 1.6	NS
Pregnancy complications, *n*. (%)	25 (29.4%)	16 (38.1%)	NS
Vaginal delivery, *n*. (%)	57 (67.1%)	22 (52.4%)	NS
Cesarean section, *n*. (%)	28 (32.9%)	20 (47.6%)	NS
Spontaneous vaginal delivery, *n*. (%)	55 (64.7%)	20 (47.6%)	NS
Vacuum-assisted vaginal delivery, *n*. (%)	2 (2.4%)	2 (4.8%)	NS
Elective cesarean section, *n*. (%)	14 (16.5%)	9 (21.4%)	NS
Urgent cesarean section, *n*. (%)	14 (16.5%)	11 (26.2%)	NS
Inductions, *n*. (%)	26 (30.6%)	8 (19.0%)	NS
Epidural anesthesia, *n*. (%)	24 (28.2%)	10 (23.8%)	NS
Meconium-stained amniotic fluid 3rd degree, *n*. (%)	1 (1.2%)	0	NS
Shoulder dystocia	0	0	NS
Perineal laceration 3rd degree, *n*. (%)	1 (1.2%)	0	NS

*Data are presented as mean (± SD) or as number of women (%)*.

*p: NS = not significant, < 0.05 = significant*.

*SSRIs, Selective Serotonin Reuptake Inhibitors*.

*Pregnancy complications: pPROM, IUGR, Hyperemesis, Cholestasis of pregnancy, Polyhydramnios, Oligohydramnios, Anhydramnios, Pregnancy Induced Hypertension, Gestational Diabetes*.

*Complications at delivery: perineal lacerations III or IV degree, meconium stained amniotic fluid, shoulder dystocia*.

The SSRIs group presented statistically relevant higher rates of low birth weight newborns (14.3%, *p* = 0.01) and significantly lower Apgar scores at 1 and 5 min after delivery (*p* = 0.00 and *p* = 0.02). Higher scores were more prevalent in *controls* than in *cases*, whereas the lowest scores were registered only in *cases* (*p* = 0.01). Neonatal sex was not a discriminant factor (Table 3). Incidence of other neonatal complications in the two groups was not statistically relevant.

**Table 3:** Neonatal outcomes.

	**Controls newborns (*n* = 85)**	**SSRIs newborns (*n* = 42)**	***p-*value**
Neonatal sex			NS
Female	41 (48.2%)	22 (52.4%)	
Male	44 (51.8%)	20 (47.6%)	
Preterm	4 (4.7%)	3 (7.1%)	NS
Neonatal weight (gr)	3303.8 ± 431.0	3148.9 ± 593.5	NS
LBW	1 (1.2%)	6 (14.3%)	0.011
APGAR score at 1′ mean	9.7 ± 0.8	9.0 ± 1.3	0.006^*^
APGAR score at 1′			0.017*^†^*
6	0	3 (7.1%)	
7	2 (2.4%)	4 (9.5%)	
8	3 (3.5%)	5 (11.9%)	
9	12 (14.1%)	8 (19.0%)	
10	68 (80.0%)	22 (52.4%)	
APGAR score at 5′ mean	10 ± 0.2	9.7 ± 0.6	0.023^*^
APGAR score at 5′			0.013*^†^*
8	0	3 (7.1%)	
9	3 (3.5%)	6 (14.3%)	
10	82 (96.5%)	33 (78.6%)	
APGAR score at 1′ <7	0	3 (7.1%)	0.069
UA pH	7.3 ± 0.1	7.3 ± 0.1	NS
UA BE	−2.9 ± 3.6	−2.1 ± 4.9	NS

*Data are presented as mean (± SD) or as number of women (%)*.

*SSRIs, selective serotonin reuptake inhibitors*.

*Preterm, newborns of < 37 gestational age*.

*LBW, low birth weight. Birth weight of a liveborn infant of <2,500 g regardless of gestational age*.

*UA, umbilical artery; BE, base excess*.

* *Student's t-test*.

† *Pearson's chi-squared test (χ^2^)*.

Table 4 presents neonatal outcomes in relation to the maternal and fetal genotypes. No significant difference was observed in neonatal outcomes in relation to either genotype.

**Table 4:** Neonatal outcomes considering maternal and fetal genotype.

**Maternal genotype**	**Normal (*n* = 12)**	**Altered (*n* = 18)**
Gestational age at delivery (week)	39.4 ± 1.7	38.9 ± 1.6
Preterm	1 (8.3%)	1 (5.6%)
APGAR sore at 1′ <7	2 (16.7%)	0
APGAR score at 5′ <7	0	0
Neonatal complications	9 (75.0%)	8 (44.4%)
Neonatal weight (gr)	3316.9 ± 667.6	3236.9 ± 579.5
LBW	1 (8.3%)	2 (11.1%)
UA ph	7.31 ± 0.1	7.29 ± 0.1
UA BE	−2.9 ± 3.1	−1.8 ± 7.2
**Fetal genotype**	**Normal (*****n*** **=** **10)**	**Altered (*****n*** **=** **22)**
Gestational age at delivery mean (week)	39.1 ± 1.9	39.1 ± 1.4
Preterm	1 (10.0%)	1 (4.5%)
APGAR score at 1′ <7	1 (10.0%)	1 (4.5%)
APGAR score at 5′ <7	0	0
Neonatal complications	8 (80.0%)	9 (40.9%)
Neonatal weight (gr)	3065.0 ± 599.9	3355.7 ± 583.1
LBW	2 (20.0%)	1 (4.5%)
UA ph	7.3 ± 0.1	7.3 ± 0.1
UA BE	−4.1 ± 4.6	−1.3 ± 5.8

*Data are presented as mean (± SD) or as number of women (%)*.

*Preterm: newborns of < 37 gestational age*.

*Neonatal complications: Low Birth Weight, a birth weight of a liveborn infant of <2,500 g regardless of gestational age; Small for Gestational Age, infants with a birth weight below the 10th percentile for gestational age; APGAR scores <7; neonatal breathing problems; neurologic symptoms; respiratory distress syndrome; access to neonatal intensive care unit; persistent pulmonary hypertension of the newborn; poor neonatal adaptation syndrome; neonatal abstinence syndrome; transient tachypnea of the newborn*.

*LBW, low birth weight. Birth weight of a liveborn infant of <2,500 g regardless of gestational age*.

*UA, umbilical artery; BE, base excess*.

## Discussion

This study shows that newborns exposed to SSRIs during fetal life are at increased risk of poor neonatal outcomes, in terms of low birth weight, low Apgar scores, and poor neonatal adaptation syndrome. The case-control population showed similar demographic, anthropometric and socio-economic features, except for smoking. Higher rates of smoking among depressed women, compared to women without a psychiatric diagnosis, were reported in previous studies (20–23). It is not clear if smoking may be related with depression. Levels of dopamine are often low in people with depression, and these individuals may then use cigarettes as a way of temporarily increasing their dopamine supply, in order to increase pleasurable feelings (24).

The distribution of the psychiatric diagnosis in this study was in line with epidemiological data (25). Depressive disorders accounted for 43% of the diagnosis, followed by anxiety disorders (26%) and mixed anxiety-depressive disorder (26%). Only 5% of patients had a bipolar diagnosis. Also the use of drugs was in line with epidemiological data with sertraline as the most frequent drug (26). This is because of its short half-life and consequent easier use and safer profile in pregnancy.

Neonatal sex was not a discriminant factor nor a bias for the results. Low birth weight newborns were significantly more frequent in the SSRIs group compared to the control (14.3 vs. 1.2%). These findings are in line with previous studies, reporting a significant association between antidepressant use in pregnancy and LBW and PTB (5, 27–29). Both PTB and LBW can also be a consequence of the underlying psychiatric disorder (4, 7, 20–22, 30). However, most studies used prescription and pharmacy dispensation data to classify exposed and not exposed, but the adherence to the prescribed treatment was unknown (30). Moreover, most studied retrospective data from national registers (20, 21, 30) and others did not consider the degree of the underlying psychiatric disorder (30). These limitations are not present in this study.

Smoking may be a confounder of this result, since it can cause LBW (31, 32) and in this study smoking rates were relevantly different between groups.

In this study, Apgar scores at 1 and 5 min after delivery were significantly lower in the SSRIs group. Besides averages, differences in APGAR score distribution were more significant: higher scores were more prevalent in controls than in cases, whereas the lowest scores were registered only in cases (*p* = 0.01). Clinically, these newborns experienced symptoms related to poor neonatal adjustment syndrome (PNAS), respiratory distress syndrome (RDS) or transient tachypnea of the newborn (TTN). PNAS was clinically defined as Finnegan score ≥ 8 in three consecutive measurements in newborns with suggestive clinical presentation (i.e., jitteriness, poor muscle tone, weak cry, respiratory distress, hypoglycemia, low Apgar scores, and seizures) (33). It accounted for 56% of newborns and was the most represented adverse outcome. The most represented symptoms (47%) were the neurological ones including: tremors, irritability, hyper-hypotonus, hyperreflexia, jitteriness, restlessness. Respiratory distress requiring oxygen supplementation was present in 5 newborns, none manifested pulmonary hypertension or seizures. All these side effects were transient and resolved within a couple of months. Seven newborns required admission in the neonatal ward.

It is unclear what causes PNAS. The fact that SSRIs cross the placenta may be an underlying pathophysiologic mechanism through possible increases in serotonin concentrations in the developing fetus. Increased serotonin concentrations may in turn impact on fetal cardiovascular, respiratory and neurological development. Accumulation of the antidepressant in the neonate could lead to serotonergic toxicity. Finally, the abrupt discontinuation of neonate exposure to the antidepressant occurring at delivery may cause withdrawal signs (34).

These findings are in agreement with recent studies showing that treatment of maternal psychiatric disorders with SSRIs during pregnancy is related to higher risk of respiratory distress or neonatal maladaptation. In particular, higher risk of neonatal problems, low Apgar scores, breathing problems, access to neonatal care unit, and longer hospital staying have been reported (21, 34–41). However, some of these studies had a number of limitations in the study design and in the selection of the included population: most studies were retrospective (38) or drew data from several national registers (36); others collected data from different hospitals (36, 38) and considered a not homogenous population (38). Finally, some studies did not take into account confounding variables, as the underlying maternal psychiatric condition, potentially leading to misclassification bias (40), or maternal Body Mass Index (BMI) (36).

These limitations were not present in this study, since data were prospectively collected in one single hospital with the same procedures and the same multidisciplinary medical team. Moreover, the population of this study was homogeneous and evaluated for confounding variables, as the underlying psychiatric diagnosis and maternal BMI. These were main strengths of this study. Another point of strength of this study was that the analysis of neonatal outcomes included anthropometric and short-term clinical outcomes, and their relation with maternal and fetal pharmacogenetic variables.

Indeed, maternal and fetal genotypes of genes involved in SSRIs metabolism were evaluated in relation to neonatal outcomes. Due to little sample size, we did not find significant differences. However, as we previously reported, we observed three *cases* with an altered polymorphism that was associated to an altered pharmacokinetic resulting in neonatal toxicity (18). Our hypothesis is that maternal and fetal functional polymorphisms in genes involved in SSRIs metabolism could affect their pharmacokinetics—how much drug is transferred to the fetus throughout the whole pregnancy and at delivery according to umbilical/plasma ratio—resulting in different drug exposure, clinical SSRI maternal responsiveness, and neonatal adverse outcomes. Given the high number of polymorphisms involved in SSRIs metabolism, a study with a larger sample size and adequate power is needed to draw conclusions that may be relevant in clinical practice.

Several limitations do exist in this study. First, the low sample size, but, on the other hand the main strength of this study is the prospective approach. Than, the presence of major confounders such as smoking, that was a relevant variable in this *case-control* population. Nevertheless, it is well-known that smoking is much more prevalent in women with depressive disorders in respect to not depressed ones.

With regard to the pharmacogenetic aspect, only the major causal variants in CYPs gene were taken into account, without considering other genetic variants described to influence CYPs expression and activity. Also, the genetic variability was not explored, due to the differences in transporter-dependent SSRI reuptake efficiency. Moreover, a control group of depressed-not-treated women was not considered. In a causal discussion, we could not ascertain that the differences found were due to SSRI treatment and not to the underlying psychiatric illness. Finally, drug compliance was evaluated during obstetric and psychiatric visits every month, but real compliance could not be determined in all cases.

## Conclusions

This study confirms and expands previous data that correlate SSRIs use during pregnancy to poor neonatal outcomes in terms of LBW, low Apgar scores and, clinically, to PNAS. However, poor neonatal outcome may also be related to untreated psychiatric diseases. Therefore, identification of infants at risk of PNAS is critical to child management since it could significantly influence neonatal outcomes.

## Ethics Statement

This study was carried out in accordance with the recommendations of the Medical Ethics Committee of Sacco Hospital, Milan, Italy, with written informed consent from all subjects. All subjects gave written informed consent in accordance with the Declaration of Helsinki. The protocol was approved by the Medical Ethics Committee of Sacco Hospital, Milan, Italy.

## Author Contributions

SCo and MM: patients' enrollment and follow up, maternal data and specimen collection, maternal and neonatal data analysis and interpretation, manuscript draft elaboration. PP: maternal and fetal data analysis and interpretation, manuscript revision. CM: statistical data analysis and interpretation. AM: neonatal data collection, evaluation, and interpretation. DC, SCh, and SB: genotyping. LP: neonatal data evaluation and interpretation, manuscript revision, and approval of final version. EC: manuscript revision and approval of final version. IC: study conception and design, manuscript revision, and approval of final version.

### Conflict of Interest Statement

The authors declare that the research was conducted in the absence of any commercial or financial relationships that could be construed as a potential conflict of interest.

## Abbreviations

BMI: Body Mass Index
CYP: cytochrome P
LBW: low birth weight
OR: odds ratio
PNAS: poor neonatal adaptation syndrome
PPHN: Persistent Pulmonary Hypertension of the Newborn
PTB: preterm baby
RDS: respiratory distress syndrome
SD: standard deviation
SGA: small for gestational age
SSRIs: selective serotonine inhibitors
TDM: therapeutic drug monitoring
TTN: Transient Tachypnea of the Newborn.
